# Epidemiology and outcomes of out-of-hospital cardiac arrests at sport and recreational events in England, 2015–2022

**DOI:** 10.1016/j.resplu.2025.101168

**Published:** 2025-11-19

**Authors:** Helen Winterburn, Gavin D. Perkins, Chen Ji, Scott Booth, Adam de Paeztron, Rachael Fothergill, Terry P. Brown

**Affiliations:** a Warwick Medical School, University of Warwick, CV4 7AL, UK; b London Ambulance Service NHS Trust, SE1 8SD, UK; c Applied Research Collaboration West Midlands, Warwick Clinical Trials Unit, University of Warwick, CV4 7AL, UK

**Keywords:** Out-of-hospital cardiac arrest, Sport, Recreation, Epidemiology, Cardiac Arrest Registry

## Abstract

**Background:**

Previous studies suggest favourable outcomes for patients experiencing exercise-related out-of-hospital cardiac arrest (OHCA). This study aimed to examine the characteristics and outcomes of OHCAs occurring at sports and recreational events in England.

**Methods:**

Data were obtained from the OHCA Outcomes registry for cases between 2015 and 2022 where resuscitation was attempted. Descriptive statistics were used to compare OHCA characteristics with outcomes: return of spontaneous circulation (ROSC) sustained to hospital handover and survival (discharge or 30-day). Logistic regression identified factors influencing outcomes.

**Results:**

Of EMS-resuscitated OHCAs, 0.5 % (N = 1316) occurred at sports and recreational events. ROSC sustained to hospital handover occurred in 43 % of cases, and 25 % survived. Most patients were male and over 65-years. These OHCAs were more likely to be witnessed (73.5 %), receive bystander cardiopulmonary resuscitation (CPR) (91.8 %), have an automated external defibrillator (AED) attached (34.3 %) and receive an ambulance response within 7-min (43.5 %). Survival was 36 % in cases that were bystander witnessed with an initial shockable rhythm. ROSC to hospital handover improved survival further (93.6 %). Regression analysis indicated initial shockable rhythm had the greatest impact on outcomes (ROSC: odds ratio (OR) = 2.97, 95 %CI = 2.25–3.92; survival: OR = 4.25, 95 %CI = 2.96–6.10).

**Conclusion:**

OHCA at sports/recreational events are rare but show better outcomes than the general population, driven by higher rates of witnessed arrests, bystander interventions and initial shockable rhythms. Findings underscore the importance of strengthening the chain of survival through early recognition, CPR training, and AED.

## Highlights

• Out-of-hospital cardiac arrest (OHCA) at sports and recreational events in England is a rare phenomenon.
• OHCAs at such events experience favourable bystander interventions; cardiopulmonary resuscitation (CPR) and use of an automated external defibrillator (AED).
• Survival rates are significantly higher than in the general population.
• Highlights importance of optimising the chain of survival: recognition of a cardiac arrest and CPR training, and access to a nearby AED.

## Introduction

An out-of-hospital cardiac arrest (OHCA) is a critical public health concern that can have devastating consequences. Although rare, OHCAs at sports and recreational events pose a serious and often underestimated risk. While the risk is often perceived as low in young, healthy individuals, various factors—including underlying heart conditions, environmental stressors, and physical exertion—can significantly increase the likelihood of an event.^1^ The importance of a rapid response and effective intervention cannot be overstated, as timely cardiopulmonary resuscitation (CPR) and the use of automated external defibrillators (AEDs) can dramatically improve survival rates.^2^, ^3^

OHCAs occurring whilst people are exercising or playing sport, or even spectating, often receive media coverage which may cause concern amongst the public as sports people are viewed as the healthiest in society.^4^ Events in recent years in Europe and USA have brought the ‘sports paradox’ to media and public attention.^5^, ^6^, ^7^, ^8^ The phenomenon is that for health benefits, exercise/sport is rightly promoted, whilst conversely for some people in certain situations vigorous activity could increase transient cardiac event risk.^9^, ^10^ OHCAs among spectators also receive publicity, where factors such as demographics, physical and emotional stress, substance abuse and meteorological conditions increase the risk of an OHCA.^11^

According to the OHCA Outcomes registry English ambulance services responded to over 98,000 calls to attend an OHCA in 2022, about a third of which were treated by emergency medical services (EMS).^12^ Based on the Utstein location, 0.9 % occurred in event locations recorded as sports/recreational. However, these high-level data do not provide specific information on the epidemiology or outcomes of OHCAs occurring at such events. Therefore, the aim of this study was to determine the characteristics and outcomes of OHCAs occurring at sports/recreational events in England, and to ascertain what factors could inform public awareness campaigns or emergency service resource planning in order to improve survival rates.

## Methods

### Study design and setting

This is a retrospective analysis of OHCAs where EMS continued or commenced resuscitation, that occurred between 1st January 2015 and 31st December 2022. Information was obtained from OHCA Outcomes (OHCAO) registry, based at the University of Warwick’s Clinical Trials Unit.^13^

### Study population

This study included patients of all ages and all aetiologies where the OHCA was diagnosed and confirmed by EMS.

### Data collection and definitions

The registry has two variables from which we could identify cases. The Utstein arrest location where the event occurred or the patient was found.^14^ The EMS, or OHCA, location where ambulance services provided the address of where the OHCA occurred, which could be an address with or without a postcode, just a postcode, or What-3-Words. Cases were identified by searching for any Utstein location recorded as a sport or recreational event and the EMS location using terms that might indicate a sport or recreational event (see Supplementary Table 1) as some services do not report Utstein location. Identified cases were manually checked to ensure the location fitted the criteria. In this study we were not able to discern whether the OHCA patient was participating in physical activity or was a spectator. Information abstracted included: patient data, event data, primary assessment, and outcomes.

Age at date of OHCA was calculated from the patient’s date of birth and incident date. According to the updated Utstein definition, unknown initial aetiology was recorded as a medical cause (presumed).^14^, ^15^ The initial cardiac rhythm was defined into two different classifications: (1) shockable rhythm included ventricular fibrillation (VF) and pulseless ventricular tachycardia (VT); (2) non-shockable rhythm included asystole, pulseless electrical activity, and bradycardia or other/unknown.

### Outcome measures

The primary outcomes of interest were ROSC sustained to hospital handover and survival. Up until 2020 survival was defined as survived to hospital discharge, after which it was defined as survived to 30-days. Both have been observed to be equivalent survival metrics.^16^

### Statistical analysis

Descriptive analysis was used, where appropriate, via Chi-square analysis with Fisher’s exact test and *t*-test to compare characteristics of the OHCA against outcomes. The cohort was split into three age-groups: ≤35-years (≤35 y), >35-years to ≤65-years (>35–≤65 y) and >65-years (>65 y). The younger age-group was selected as a veteran athlete is usually defined as an individual >35 y,^17^ and the older age-group anyone greater than the median age of the cohort. Any missing data was classified as unknown. Univariate logistic regression analysis was performed to examine the association between individual OHCA characteristics and outcomes. Any variable with a p-value ≤0.2 was integrated into the final model. A backwards stepwise regression model was then developed, using a significance level of p > 0.05 for removal from the model. Before running the model, the variables were tested for multi-collinearity using pairwise correlation. Goodness-of-fit of the model was assessed by the Hosmer-Lemeshow test. All analyses were conducted in STATA version 18 (Stata Corp, USA).

### Ethics

The study was approved by the University of Warwick’s Biomedical and Scientific Research Ethics Committee (BSREC 14/22-23). Ethical approval for the OHCAO project was gained from the National Research Ethics Committee South Central, reference number 13/SC/0361. Confidential Advisory Group approval has been granted, reference numbers 22CAG0072 and 22CAG0087, to collect identifiable patient information where it is not practical to obtain consent.

## Results

### Descriptive statistics

**Fig. 1:**
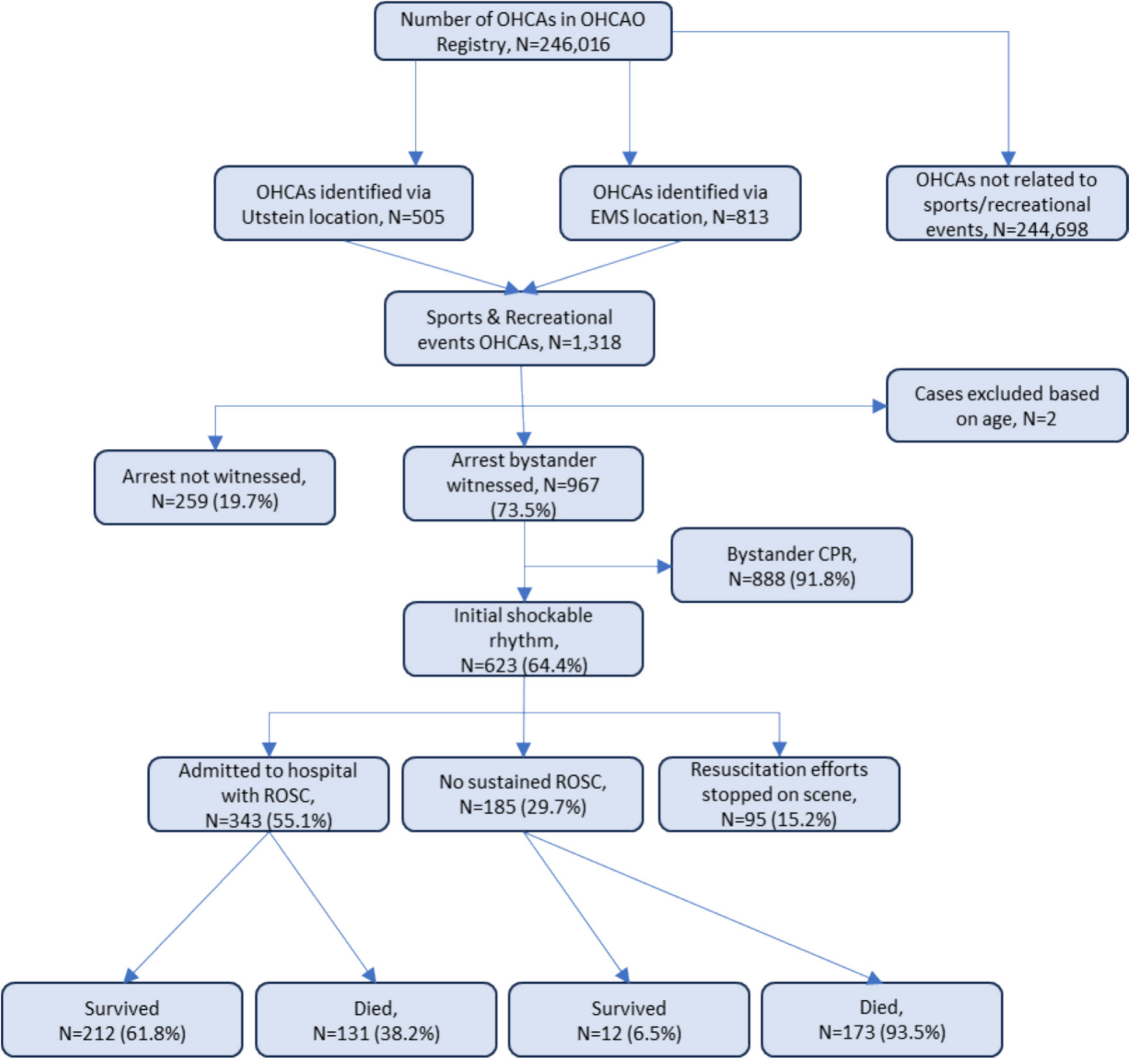
Flow chart for identification of out-of-hospital cardiac arrests (where resuscitation continued or commenced by emergency medical services) at sports and recreational events in England, 2015–2022.

Between 2015 and 2022 details of 246,016 OHCAs, where EMS continued or commenced resuscitation, were submitted to the OHCAO registry. A total of 1318 cases were identified as occurring at a sports or recreational event (Fig. 1); 0.5 % of all EMS-resuscitated OHCAs. Two cases were excluded because their age was greater than the eldest person living in England. Based on 2018 mid-year population for England (55.98 million)^18^ the incidence is around 0.3/100000-persons/year. Table 1 gives the characteristics of this cohort of patients and compares the different age groups.

**Table 1:** Characteristics of out-of-hospital cardiac arrests occurring at sports and recreational events in England, 2015–2022 (number and percentage unless otherwise stated).

	**Total** **(N = 1316)**	**Age Group (years)**			
		**≤35 (N = 97)**	**35–≤65 (N = 536)**	**>65 (N = 642)**	**p-value**
**Proportion male**	1152 (87.5)	74 (76.3)	479 (90.9)	570 (89.9)	0.001

**Age (years):**	(n = 1275)				
Mean(SD^a^)	62.5 (16.6)	23.6 (9.0)	54.4 (7.7)	75.1 (6.8)	<0.001
Median (IQR^b^)	65.2 (54.0–74.1)	25.8 (17.9–31.0)	56.0 (49.1–61.0)	74.0 (69.4–80.0)	<0.001

**Witness:**					
Unwitnessed/Unknown	259 (19.7)	28 (28.9)	96 (17.9)	124 (19.3)	0.04
Bystander	967 (73.5)	56 (57.7)	400 (74.6)	482 (75.1)	0.001
EMS	90 (6.8)	13 (13.4)	40 (7.5)	36 (5.6)	0.02

**Bystander CPR rate:**					
All cases	1085 (82.5)	68 (70.1)	452 (84.3)	535 (83.3)	<0.001
Unwitnessed	189 (73.0)	17 (60.7)	77 (80.2)	89 (71.8)	0.09
Bystander witnessed	888 (91.8)	51 (91.1)	371 (92.8)	442 (91.7)	0.81

**AED Attached**	452 (34.3)	31 (32.0)	162 (40.5)	188 (39.0)	0.69
**Initial aetiology:**					
Medical	1186 (90.1)	65 (67.0)	494 (92.2)	594 (92.5)	<0.001
Trauma	42 (3.2)	13 (13.4)	16 (3.0)	11 (1.7)	<0.001
Drowning	25 (1.9)	7 (7.2)	6 (1.1)	10 (1.6)	<0.001
Asphyxia	16 (1.2)	3 (3.1)	7 (1.3)	5 (0.8)	0.13
Other/Unknown	47 (3.6)	9 (9.3)	13 (2.4)	22 (3.4)	0.003

**Initial shockable rhythm**	740 (56.2)	48 (49.5)	312 (58.2)	359 (55.9)	0.26

**Ambulance response time (min):**	(n = 1238)	(n = 92)	(n = 507)	(n = 601)	
Mean (SD^a^)	9.3 (11.1)	7.3 (4.5)	9.7 (12.9)	9.2 (10.4)	0.09
Median (IQR^b^)	7.6 (5.0–11.3)	6.7 (4.7–9.9)	7.7 (4.9–11.6)	7.9 (5.0–11.3)	0.07
<7 min (N, %)	573 (43.5)	54 (55.7)	237 (44.2)	264 (41.1)	0.03

**Utstein comparator group**^c^	623 (47.3)	34 (35.1)	265 (49.4)	305 (47.5)	0.03

a Standard deviation.

b Inter-quartile range.

c Bystander witnessed and initial rhythm is shockable.

Only 7.6 % of cases, with a confirmed age, occurred in those aged ≤ 35y. Approximately 87.5 % of cases were male; however, there was a significant difference between the three age-groups (≤35y-76.3 %; >35y–≤65 y-90.9 %; >65 y-89.9 %; p = 0.001). The median age was 65.2-years (IQR: 54.0–74.1 y). Almost three-quarters of cases were witnessed by a bystander (73.5 %), with significantly more in the older age-groups (74.6 % and 75.1 % vs 57.7 %; p = 0.001).

Bystander CPR in unwitnessed and bystander witnessed cases was high at 73.0 % and 91.8 %, respectively. Similarly, high bystander CPR rates were observed in all age groups. Application of an AED was high at around 34 %. In the younger age group, a significantly higher proportion of OHCAs were due to trauma (≤35y:13.4 %; >35–65y:3.0 %; >65y:1.7 %; p < 0.001) and drowning (≤35y:7.2 %; >35–≤65y:1.1 %; >65y:1.6 %; p < 0.001), whereas fewer were attributed to medical reasons (≤35y:67.0 %; >35–≤65y:92.2 %; >65y:92.5 %; p < 0.001) compared to the older age groups. An initial shockable rhythm was detected in around 56 % of all cases (≤35y:49.5 %; >35–≤65y:58.2 %; >65y:55.9 %). Median ambulance response time was 7.6 min (IQR: 5.0–11.3-min); about 44 % of cases being reached in under 7-min. Significantly more cases in the ≤35 y group were reached in <7-min (55.7 % vs. 44.2 % and 41.1 %; p = 0.03). A significantly smaller proportion of cases in the younger age group met the Utstein comparator group criteria, defined as a bystander-witnessed arrest with an initial shockable rhythm (35.1 % vs 49.4 % and 47.5 %; p = 0.03).

ROSC was achieved at any time in 647 (49.2 %) of cases, with similar levels in all age groups. (Table 2). A total of 562 (42.8 %) cases sustained a ROSC to hospital handover, with similar proportions in all age groups. A total of 328 (24.9 %) survived (≤35y = 30.9 %; >35–≤65y = 28.4 %; >65y = 22.4 %; p = 0.03). Survival in the Utstein comparator group was 36.0 % overall, and significantly higher amongst the ≤ 35y group compared to the older ones (52.9 % vs 38.9 % and 33.1 %; p = 0.05).

**Table 2:** Outcomes of out-of-hospital cardiac arrests at sports and recreational events in England, 2015–2022 (number and percentage).

	**Total** **(N = 1316)**	**Age group (years)**			
		**≤35 (N = 97)**	**35–≤65 (N = 536)**	**>65 (N = 642)**	**p-value**
ROSC at any time	647 (49.2)	47 (48.5)	277 (51.7)	310 (48.3)	0.49
ROSC sustained to hospital handover	562 (42.7)	44 (45.4)	239 (44.6)	267 (41.6)	0.53
Survived^a^	328 (24.9)	30 (30.9)	152 (28.4)	144 (22.4)	0.03
Utstein Comparator Group^a^ survival	224 (36.0)	18 (52.9)	103 (38.9)	101 (33.1)	0.05

^b^ Bystander witnessed and initial rhythm is shockable; see Table 1 for number of cases.

a To hospital discharge or 30-days.

Table 3 presents a comparison of OHCA cases where a ROSC was sustained to hospital handover and the patient survived. Survival was significantly more likely when the arrest was witnessed by a bystander, CPR was provided by a bystander, and the initial rhythm was shockable—characteristics typical of the Utstein comparator group.

**Table 3:** Characteristics of out-of-hospital cardiac arrests occurring at sports and recreational events in England, 2015–2022 that sustained a ROSC to hospital and survived (number and percentage unless otherwise stated).

	**ROSC sustained to hospital handover**			**Survived**		
	**Yes**	**No**	**p-value**	**Yes**	**No/Unknown**	**p-value**
**Number**	562 (42.7)	754 (57.3)		328 (24.9)	988 (75.1)	

**Witness:**						
Unwitnessed/Unknown	60 (10.7)	199 (26.4)	<0.001	24 (7.3)	235 (23.8)	<0.001
Bystander	453 (80.6)	514 (68.2)	<0.001	266 (81.1)	701 (71.0)	<0.001

**Bystander CPR**^a^	423 (93.4)	465 (90.5)	0.100	247 (92.9)	641 (91.4)	0.45
**AED attached**^a^	195 (43.1)	189 (36.8)	0.046	117 (44.0)	267 (38.1)	0.09

**Initial aetiology:**						
Medical	525 (93.4)	661 (87.7)	<0.001	315 (96.0)	871 (88.2)	<0.001
Trauma	9 (1.6)	33 (4.4)	0.004	3 (0.9)	39 (3.9)	0.007
Drowning	8 (1.4)	17 (2.3)	0.24	1 (0.3)	24 (2.4)	0.02

**Initial shockable rhythm**	413 (73.5)	327 (43.4)	<0.001	274 (83.5)	466 (47.2)	<0.001
**Utstein comparator group**^b^	343 (61.0)	280 (37.1)	<0.001	224 (68.3)	399 (40.4)	<0.001
**Sustained ROSC**				307 (93.6)	255 (25.8)	<0.001

a Bystander witnessed cases only.

b Bystander witnessed and initial rhythm is shockable.

Amongst cases that survived, 93.6 % (n = 307) had sustained a ROSC to hospital handover, whereas 25.8 % of those that did not had (Table 3; p < 0.001). The odds of survival in the sustained ROSC group was 23.7 times higher (OR = 23.7; 95 % CI: 14.8–37.8). Among patients who did not sustain ROSC, survival was still twice as likely if the initial rhythm was shockable (6.4 %, 14/220) compared to non-shockable rhythms (3.3 %, 7/214).

### Regression analysis

Logistic regression analysis highlighted significant independent associations with the observed outcomes (Supplementary Table 2). Table 4 gives the multivariable logistic regression models for each outcome, adjusted for age, sex and other variables in model. Three factors consistently showed a significant positive impact on outcomes: having of an AED attached, an initial shockable rhythm, and an ambulance response time under seven minutes. These factors were associated with an increased chance of sustaining ROSC to hospital handover and overall survival. In addition, bystander witness had a significant on survival. Bystander CPR did not appear to have a statistically significant role when controlled for other factors. Bystander CPR rates were high across the cohort (see Table 1). Similar rates were observed regardless of whether a ROSC was sustained to hospital handover, or the patient ultimately survived.

**Table 4:** Multivariable logistic regression models for return of spontaneous circulation (ROSC) sustained to hospital and survival^a^ in non-EMS witnessed out-of-hospital cardiac arrest occurring at sports or recreational events in England, 2015–2022 (N = 1097).

	**ROSC sustained to hospital handover**			**Survival**^a^		
	**Odds ratio**^b^	**p-value**	**95 % CI**	**Odds ratio**^b^	**p-value**	**95 % CI**
Bystander witnessed	1.33	0.36	0.72, 2.44	2.27	0.03	1.08, 4.77
Bystander CPR^c^	1.50	0.15	0.87, 2.59	0.99	0.98	0.54, 1.83
AED attached^c^	1.42	0.02	1.06, 1.89	1.38	0.05	1.00, 1.91
Shockable rhythm	2.97	<0.001	2.25, 3.92	4.25	<0.001	2.96, 6.10
Ambulance response time ≤7 min (yes)	1.79	<0.001	1.38, 2.33	2.11	<0.001	1.56, 2.84

a to hospital discharge or 30-days.

b Odds ratios adjusted for age (continuous) and sex plus other variables listed.

c Bystander witnessed cases only.

## Discussion

We have shown that OHCAs at sports or recreational events in England are rare, accounting for 0.5 % of all cases where resuscitation was attempted. The observed survival rate of 24.9 % is more than double that of OHCAs in all locations in England, around 9.0 %.^19^ Sustaining ROSC to hospital handover was significantly associated with the arrest being witnessed, the presence of an initial shockable rhythm, the administration of bystander CPR, and the use of an AED in bystander-witnessed cases (Table 3). An ambulance response time within the national standard for category one calls (<7 min) also significantly improved outcomes.

The proportion of cases in which resuscitation was started was similar to that previously reported in London (0.5 %).^20^ Our figure is, however, lower than that seen in Japan,^21^ North Holland,^22^ Australia,^23^ Denmark^24^ and Norway.^25^ Our case identification was based on the agreed Utstein location definition,^14^, ^15^ supplemented by searching the EMS location using an agreed list of search terms (Supplementary Table 1). Nevertheless, this list may not have been comprehensive, and we could still have missed a number of cases. Also, some ambulance services only provide the location postcode or Utstein style location data, and it is difficult to determine the building type at that location.

The mean and median ages in the cohort were 62.5y and 65.2y, respectively – both younger than the national average.^19^ Notably, the mean age was higher than reported in previous studies,^20^, ^23^, ^26^ while the median age lower than that observed from Denmark and Japan.^21^, ^24^ Our cohort most likely included both participants and spectators, the latter possibly being older than the former; we could not distinguish between the two from the data available on the registry. The majority of cases involved male patients, consistent with findings from other studies. This may be attributed to higher participation in exercise and sports among adult men, as well as their greater representation among spectators at most sporting events.^27^

About 90 % of OHCAs were of medical aetiology. However, in the ≤ 35y age group significantly more OHCAs were as a result of trauma, drowning and asphyxia compared to the older age groups, possibly reflecting the type of event this age group participate in (Table 1). Similar differences for trauma and drowning have also been observed in Sweden.^28^

OHCAs with an initial shockable rhythm have been recorded as having better survival chances,^29^ but VF deteriorates rapidly without any intervention, highlighting the need for early CPR and defibrillation.^30^ Our analysis has shown an initial shockable rhythm was the main factor affecting all outcomes (Table 3 and Supplementary Table 3), similar to Australia.^23^ We observed an increased number of cases with an initial shockable rhythm, compared to OHCAs in all locations (56.2 % verses 21.7 %).^19^ Our figure is well within the range (38−97 %) observed in other studies.^31^

The proportion of cases that were witnessed by a bystander was more than 20 percentage points greater than observed in the general population (73.5 % vs 50.5 %),^19^ and similar to that seen in other studies.^31^ A greater level of bystander witnessed OHCAs presumably means that there was likely earlier recognition of what was happening and prompt contact with emergency services. The bystander CPR rate in bystander witnessed cases is high at 91.8 %, which compares to 76.8 % in the general population,^19^ and similar to most other studies of sports-related OHCAs.^24^, ^31^

Consequently, there is also a significantly greater level of CPR and AED use by a bystander in this cohort (34.3 %) compared to the OHCAs that occurred in all locations (11.9 %)^19^; however, we do not know whether a shock was delivered or not. This level is similar to that observed in Australia,^23^ and notably higher than in London^20^ – which is expected given the greater number of AEDs available there. AED availability and time taken to attach AED to a patient (connection time) are important factors that impact on OHCA survival. In Italy, sports facilities with on-site AED, connection times were four-times faster than those in facilities without an AED.^32^

The importance of AED is growing significantly because it is the definitive way of re-starting the heart, and groups are being urged to purchase AEDs and make them available 24/7. The English Football Premier League have set up a fund to install AEDs and external storage cabinets at grassroots football facilities.^33^ Other schemes have been set-up by the Rugby Football Union, Rugby Football League, Lawn Tennis Association, English and Wales Cricket Board and UK Athletics. Parkrun have also made a global commitment to ensure wherever events take place the chance of survival for someone who experiences an OHCA is greatly improved by having direct access to an AED.^34^

About a quarter of all cases survived, almost three-times that seen in the general population (around 9.5 %). For Utstein comparator group cases, survival was 36.0 %, 7 % greater than that seen in general population.^19^ In the respective Utstein comparator group, survival in the ≤35 y age group was significantly higher compared to the older age groups (52.9 % vs 38.9 % and 33.1 %). In the recent systematic literature review of exercise-related sudden cardiac arrest, survival ranged from 11 % to 77 % with a median of 34 %,^31^ placing our cohort around the average. Previously, overall survival rate for exercise-related OHCAs in London was observed to be higher at about 32 %, although in the Utstein comparator group it was 42 %.^20^ As in previous studies significant improvement in survival was observed if the case was witnessed, received CPR from a bystander, an AED was used and the initial rhythm was shockable.^20^, ^24^, ^26^, ^28^, ^31^, ^35^, ^36^

Our findings highlight the importance of establishing an Emergency Action Plan at sports/recreational events to optimise the chain of survival for OHCA; such plans have been shown to be successful in high profile cases.^37^, ^38^, ^39^ The Resuscitation Council UK’s new “Field for Play” guidance recognises that anyone organising such events ensure staff are trained and AEDs are available.^40^, ^41^ All sports and exercise facilities, and recreational events, should undertake a medical risk assessment of the risk of OHCA among athletes, spectators, and staff. in settings where the risk of OHCA is elevated, staff and members should be trained to recognise and respond appropriately, including identifying cardiac arrest, initiating management, and either providing an AED directly or offering clear guidance to the nearest available device.

### Limitations

One limitation to this study was that we may have underestimated the number of OHCAs at events. We first identified cases via the Utstein location. However, the OHCAO registry recognises this to be incomplete for some ambulance services, so we used the EMS location data to identify additional cases, using specified search criteria (Supplementary Table 1). Nevertheless, the list may not have been comprehensive enough to detect these cases. Some ambulance services do not provide an address for the EMS location to enable us to use the search criteria, providing just a postcode. Second for those cases identified we could not determine whether they were participating or spectators, which may have impacted on outcomes, and also limits the extent to which we can make direct comparisons with other studies. A major limitation is that we could not determine what type of event each OHCA occurred at. It is also not clear how each ambulance service categorises the location with regards to sports/recreational event. The definition of a bystander in this study is very loose and could have been a member of the public, on-site staff trained in CPR and AED use, or member of an on-site medical team already present at the event. Information on AED availability and usage within the OHCAO registry is known to be incomplete for some ambulance services, but improving, and therefore these figures may be higher. We did not observe bystander CPR to have a statistically significant impact on outcomes; however, this was probably because bystander CPR rate was high across the board. We were limited to analysing OHCAs at sport and recreational events and therefore could not make any direct comparison with OHCAs in the home or other public places, which could be done at a later date.

### Recommendations

Our results are informative but there are gaps in the research. We recommend that work is done to distinguish between participants and spectators to determine if risk profiles and outcomes differ. Further, we will propose ambulance services improve the event type classification, to help identify high risk sports and recreational activities for focused prevention. In addition, we recommend enhancing the collection of location data to allow accurate venue identification and better epidemiological assessment. Finally, further work should be carried out on AED availability, application time, and shock delivery which are critical for improving survival rates.

## Conclusion

In conclusion, OHCAs, where EMS continue or commence resuscitation, occurring at sport and recreational events, participants, or spectators, in England is a rare event. Individuals who sustain an OHCA at such events are more likely to sustain a ROSC to hospital handover and survive (to 30-days or hospital discharge) compared to the general population. This is probably due to the OHCA is more likely to be witnessed, to have an initial rhythm that was shockable and have an AED used prior to the arrival of EMS.

## Funding

This project received no direct funding and formed part the medical degree student selected component. The Out-of-Hospital Cardiac Arrest Outcomes Registry is funded by a grant from the 10.13039/501100000274British Heart Foundation and Resuscitation Council UK

## CRediT authorship contribution statement

**Helen Winterburn:** Writing – review & editing, Writing – original draft, Visualization, Validation, Methodology, Investigation, Formal analysis, Data curation, Conceptualization. **Gavin D. Perkins:** Writing – review & editing, Supervision, Methodology, Conceptualization. **Chen Ji:** Writing – review & editing, Validation, Supervision, Methodology. **Scott Booth:** Writing – review & editing, Validation, Project administration, Investigation, Data curation. **Adam de Paeztron:** Writing – review & editing, Project administration, Investigation. **Rachael Fothergill:** Writing – review & editing, Validation, Supervision. **Terry P. Brown:** Writing – review & editing, Visualization, Supervision, Methodology, Investigation, Formal analysis, Data curation, Conceptualization.

## Funding

This study received no direct funding. The OHCAO registry received funding from the British Heart Foundation and Resuscitation Council UK.

## Declaration of competing interest

GDP is Editor-in-Chief of Resuscitation Plus and Editor of Resuscitation journals. The remaining authors declare no competing interest.
