# Supplementary material for: Epidemiology and outcomes of out-of-hospital cardiac arrests at sport and recreational events in England, 2015–2022
Source: Resusc Plus. 2025 Nov 19;27:101168. doi: 10.1016/j.resplu.2025.101168 (PMC12704064; doi:10.1016/j.resplu.2025.101168)
Supplement: Supplementary Data 2 [file mmc2.docx]

# Supplementary Tables

| Supplementary Table 1: Words searched for in the EMS location field to identify cases that occurred at sports or recreational events not defined by the Utstein location. | | | |
| --- | --- | --- | --- |
| Aquatic | Gliding | Netball | Ski |
| Athletics | Golf | Olympics | Snooker |
| Badminton | Gym | Pitch | Snow |
| Baseball | Gymnastics | Polo | Sport or Sports |
| Basketball | Hockey | Riding | Squash |
| Bowling or Bowls | Ice | Rink | Stadium |
| Boxing | Judo | Rowing | Swim or Swimming |
| Cricket | Karate | RUFC | Tennis |
| Cross-Country | Lacrosse | Rugby | Track |
| Cycle or Cycling | Leisure | Shooting | Volleyball |
| Football | Martial | Skate | Weight |
| Game | Motor | Skating | Wrestling |

| Supplementary Table 2: Univariate logistic regression for return of spontaneous circulation sustained to hospital handover(ROSC) and survival in out-of-hospital cardiac arrest occurring at sports or recreational events. | | | | | | | |
| --- | --- | --- | --- | --- | --- | --- | --- |
|  | ROSC sustained to hospital handover | | |  | Survival | | |
|  | OR | P>[Z] | CI |  | OR | P>[Z] | CI |
| Gender (male) | 1.04 | 0.85 | 0.72, 1.48 |  | 1.72 | 0.02 | 1.08, 2.75 |
| Age (years) | 0.99 | 0.09 | 0.99, 1.00 |  | 0.99 | 0.003 | 0.98, 1.00 |
| Age Group (years) | 0.91 | 0.28 | 0.76,1.08 |  | 0.77 | 0.01 | 0.63, 0.94 |
| ≤35 | Ref. |  |  |  | Ref. |  |  |
| 35-≤65 | 0.97 | 0.89 | 0.63, 1.50 |  | 0.88 | 0.61 | 0.55, 1.41 |
| >65 | 0.86 | 0.48 | 0.56, 1.32 |  | 0.65 | 0.07 | 0.40, 1.03 |
| Witness (trend): | 2.24 | <0.001 | 1.77, 2.84 |  | 2.70 | <0.001 | 2.04, 3.58 |
| Unwitnessed/Unknown | 0.33 | <0.001 | 0.24, 0.46 |  | 0.25 | <0.001 | 0.16, 0.39 |
| Bystander witnessed | 1.94 | <0.001 | 1.50, 2.52 |  | 1.76 | 0.003 | 1.29, 2.39 |
| EMS witnessed | 1.66 | 0.02 | 1.08, 2.55 |  | 2.36 | <0.001 | 1.52, 3.66 |
| Bystander CPR^1^ | 1.89 | <0.001 | 1.49, 2.41 |  | 1.65 | 0.001 | 1.24, 2.19 |
| AED use^1^ | 1.59 | <0.001 | 1.25, 2.02 |  | 1.50 | 0.003 | 1.15, 1.95 |
| Shock Rhythm | 3.62 | <0.001 | 2.86, 4.58 |  | 5.68 | <0.001 | 4.14, 7.81 |
| Ambulance response time: |  |  |  |  |  |  |  |
| Continuous | 0.96 | <0.001 | 0.94, 0.98 |  | 0.93 | <0.001 | 0.90, 0.95 |
| ≤7 minutes (yes) | 1.87 | <0.001 | 1.49, 2.34 |  | 2.31 | <0.001 | 1.77, 3.00 |
| Utstein comparator group^2^ | 2.65 | <0.001 | 2.12, 3.32 |  | 3.18 | <0.001 | 2.44, 4.14 |
| ^1^ In bystander witnessed cases  ^2^ Utstein comparator group considers cases that were witnessed by a bystander and had an initial shockable rhythm. | | | | | | | |
